# Topological Photonic Crystal Ring Resonator Pressure Sensor in the Optical Communication Range

**DOI:** 10.3390/s26020659

**Published:** 2026-01-19

**Authors:** Min Wu, Zhuoxin Yang, Hongming Fei, Han Lin

**Affiliations:** 1 College of Physics and Optoelectronics, Taiyuan University of Technology, Taiyuan 030024, China; wumin2@sxgkd.edu.cn (M.W.); yzx990921@163.com (Z.Y.); 2 College of Information Engineering, Shanxi Vocational University of Engineering Science and Technology, Jinzhong 030619, China; 3 Centre for Atomaterials and Nanomanufacturing, School of Science, RMIT University, Melbourne, VIC 3000, Australia

**Keywords:** pressure sensor, ring resonator, germanium valley photonic crystal

## Abstract

**Highlights:**

**What is the main finding?**

- An ultra-compact pressure sensor based on a valley photonic crystal topological ring resonator can achieve unidirectional transmission and high-sensitivity pressure sensing within the optical communication range.

**What is the implication of the main finding?**

- The design principle of combining material deformation and refractive index changes with topological photonic crystals can be broadly applied to design ultra-compact high-performance pressure or strain sensors.

**Abstract:**

Optical pressure sensors offer the advantages of high sensitivity, immunity to interference, and suitability for use in extreme environments. Based on the defect-immune, unidirectional transmission characteristics of valley photonic crystals (VPCs) and the refractive-index modulation of germanium under different pressures, we designed a topological ring resonator pressure sensor based on germanium VPCs. The shift of the resonance peak in the optical communication wavelength range with respect to pressure magnitude is studied to realize a pressure-sensing function. The results show that within the range of 0–10 GPa, the wavelength of the single resonance peak of the topological ring resonator pressure sensor shifts from 1580 nm to 1489 nm as the pressure increases. The sensor’s maximum detection sensitivity is 24.34 nm/GPa, and the transmittance across the bandwidth remains consistently above 0.85, with a maximum of 0.97. The germanium-based topological ring resonator pressure sensor features a compact structure with a size of 7.5 μm × 6.5 μm. It can be manufactured using existing nanofabrication technology and will have broad application prospects in the field of integrated photonic chips.

## 1. Introduction

High-performance pressure sensors exhibit characteristics such as high sensitivity, high quality factor (Q–factor), high transmission efficiency, and compactness. Currently, pressure sensors can be categorized into optical pressure sensors and electrical pressure sensors. Among them, traditional electrical pressure sensors, such as piezoresistive or capacitive sensors, offer high sensitivity but suffer from bulkiness, the need for an external power supply, and susceptibility to electromagnetic interference. Additionally, their internal resistance (especially in piezoresistive sensors) introduces significant signal noise, while limitations in materials and structures make them difficult to operate reliably in extreme environments, such as high temperatures and severe corrosion. These factors hinder the development of electrical pressure sensors [1,2]. In contrast, optical pressure sensors detect pressure by analyzing changes in the optical spectrum induced by external pressure. They offer greater design flexibility, relatively smaller size, immunity to electromagnetic interference, and the ability to operate in extreme environments. Currently, various optical pressure sensors operating in the optical communication band have been developed, including optical fiber pressure sensors [3], fiber Bragg grating pressure sensors [4], optical waveguide pressure sensors [5], and Mach–Zehnder interferometer pressure sensors [6,7]. However, traditional optical pressure sensors are generally expensive, bulky, and unsuitable for high-density photonic integration. Therefore, a fundamental breakthrough is urgently needed to design compact, small-sized pressure sensors suitable for optical communication. Some researchers have proposed using metamaterials to design micro- and nanoscale optical pressure sensors with simpler structures. However, most of these designs employ metallic materials, which suffer from intrinsic losses and exhibit low Q factors, making them unsuitable for integrated devices [8].

Pressure sensors based on photonic crystals (PCs) are highly sensitive to refractive index changes and are suitable for high-density integration. The properties of light within PCs are influenced by external parameters such as vibration, pressure, temperature, and biomolecules. The periodic modulation of the dielectric constant in PCs enables optical sensing under various environmental conditions. Optical sensors designed using various types of PC waveguides and micro-ring resonators enable high-sensitivity sensing, as their resonance peaks shift in response to changes in external conditions [9,10,11,12,13,14,15,16,17,18]. Researchers have reported a polymer-based PC pressure sensor with a size of 11 μm × 8.6 μm, achieving a resonance peak transmission of 0.97 and a sensitivity of 21 nm/GPa [17]. However, the backscattering and low transmittance of light waves in traditional PC sensors have long been critical problems to address, especially at turning points in waveguide devices, where scattering is particularly severe, leading to unnecessary losses and severely limiting sensitivity and device integration. Therefore, designing compact, high-transmission, high-sensitivity, and suitable for integration optical pressure sensors operating within the optical communication range is an important development direction. Valley photonic crystals (VPCs) based on the valley Hall effect offer advantages such as simple design, easily tunable photonic band gaps, and broad operating bandwidths, making them highly significant for integrated development and sensor applications. Topological ring resonators based on VPCs not only feature a compact structure, defect immunity, and suppressed backscattering but also enable high-quality-factor resonance peaks. These characteristics are particularly crucial for integrated sensors to achieve high transmission and high-sensitivity sensing [19,20,21,22,23,24,25,26,27,28,29,30,31,32,33,34,35,36]. However, pressure sensors based on VPCs have not yet been demonstrated.

We proposed a topological resonator pressure sensor based on a germanium-based VPC. The sensor consists of a triangular topological ring resonator and a topological straight waveguide. By simulating the band properties of topological edge states under different pressures, we investigated shifts in the VPC bandwidth within the optical communication range and variations in the transmission spectrum. Subsequently, a topological ring resonator was designed to investigate shifts in the resonance peaks in the output spectrum under different pressure conditions. The magnitude of the applied pressure is determined from the wavelength shift of the resonance peaks, thereby enabling pressure sensing. Based on this principle, a topological pressure sensor structure was designed. When the external pressure is from 0 to 10 GPa, the resonance dip wavelength of the topological ring resonator pressure sensor shifts toward higher frequency, achieving a maximum pressure detection sensitivity of 24.34 nm/GPa. The maximum unidirectional transmission in the output spectrum of the proposed ultra-compact topological resonator pressure sensor reaches 0.97, theoretically enabling high-transmission pressure sensing within the optical communication band. This topological resonator pressure sensor can be fabricated using existing micro- and nano-fabrication techniques. It not only offers a compact device structure and high transmission efficiency but also enables high-sensitivity control of resonance peaks, which is of significant importance for the development of integrated sensors.

## 2. Theoretical Model of Topological Ring Resonator Pressure Sensors

**Figure 1:**
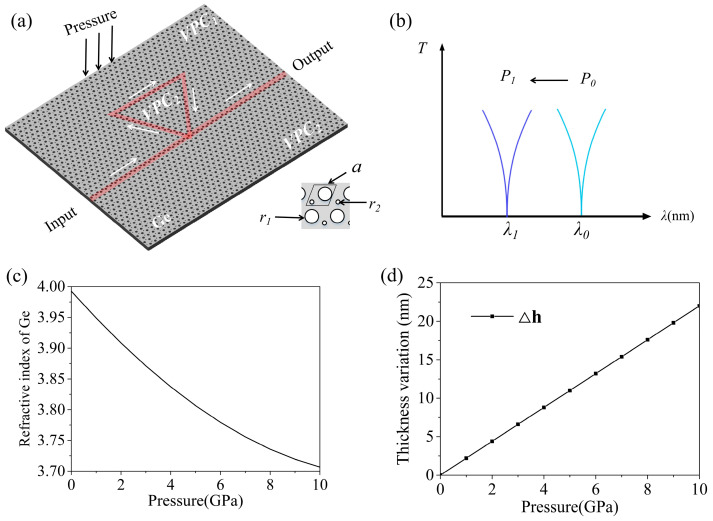
(**a**) Schematic diagram of a pressure sensor composed of a topological ring resonator coupled with a topological straight waveguide. The light source enters from the left side of the topological straight waveguide, where the arrow represents the light propagation direction in the topological pressure sensor; (**b**) When the external pressure is from *P*_0_ to *P*_1_, the wavelength of the dip moves from *λ*_0_ to *λ*_1_, where *λ*_1_ (blue) and *λ*_0_ (cyan) correspond to an applied pressure *P*_1_ and the zero-pressure condition *P*_0_, respectively; (**c**) The curve of the refractive index of germanium material versus pressure when applying pressure on the germanium plate. (**d**) The position of the resonance peak in the sensor output spectrum is fitted to a curve as pressure varies.

We presented a topological resonator pressure sensor based on a VPC, as shown in the schematic diagram in Figure 1a. The sensor structure consists of a honeycomb lattice of circular air holes embedded in a 220 nm-thick germanium substrate. During the simulation, a right-handed circularly polarized (RCP) light source is placed in one of the circular air holes in the topological straight waveguide. The arrows indicate the direction of light propagation within the topological ring resonator pressure sensor. Within the maximum pressure tolerance range of the germanium plate (less than 10 GPa), and considering the change in the refractive index of germanium under different pressures, a shift occurs in the position of the dip in the output spectrum when light passes through germanium-based topological pressure sensors with different refractive indices, thereby enabling the measurement of the corresponding external pressure. Figure 1b shows the position of one resonance dip in the output spectrum, where *λ*_1_ and *λ*_0_ correspond to an applied pressure *P*_1_ and the zero-pressure condition *P*_0_, respectively. As pressure increases, the resonance dip shifts from *λ*_0_ to *λ*_1_. The refractive index of the germanium substrate in the topological pressure sensor varies with applied pressure according to the formula [15]:

*n*_Ge_=√(15.94 − 0.36*P* + 0.0041*P*^2^) (1)

Within the pressure range of 0–10 GPa, the refractive index of germanium gradually decreases with increasing pressure, as shown in Figure 1c. Consequently, the effective refractive index of the germanium-based VPC changes, altering the resonance peak distribution in the topological sensor and thereby enabling pressure sensing. According to the formula Δ*h* = (*σ*/*E*) × *h*_0_, σ denotes the applied pressure, *E* represents Young’s modulus, and *h*_0_ is the original thickness. The thickness variation of a 220 nm germanium plate under different applied pressures can be calculated as shown in Figure 1b [37,38]. The thickness of the germanium plate under 10 GPa is 198 nm, as shown in Figure 1d. The germanium plate studied in this paper can withstand pressures up to 10 GPa (details are provided in Supplementary Materials, Section S1). By simulating the spectral response of the germanium-based VPC under different pressures, the pressure value can be characterized. Different resonance peak positions of the topological ring resonator correspond to different pressures, enabling amplified monitoring of external pressure.

The germanium-based VPC designed in this work achieves valley-momentum-locked optical transmission by breaking the spatial inversion symmetry of a honeycomb-lattice photonic crystal that possesses Dirac points in its band. This is accomplished by varying the lattice-site sizes in three directions, reducing the structural symmetry from C_6v_ to C_3v_, and lifting the degeneracy at the *K* and *K’* points [21]. The design process is divided into the following two steps: (1) First, design a germanium-based PC structure with Dirac points in the band structure. (2) Then, open a bandgap at the Dirac points by reducing the structural symmetry through modifying the lattice site sizes, thereby achieving a topological photonic bandgap.

**Figure 2:**
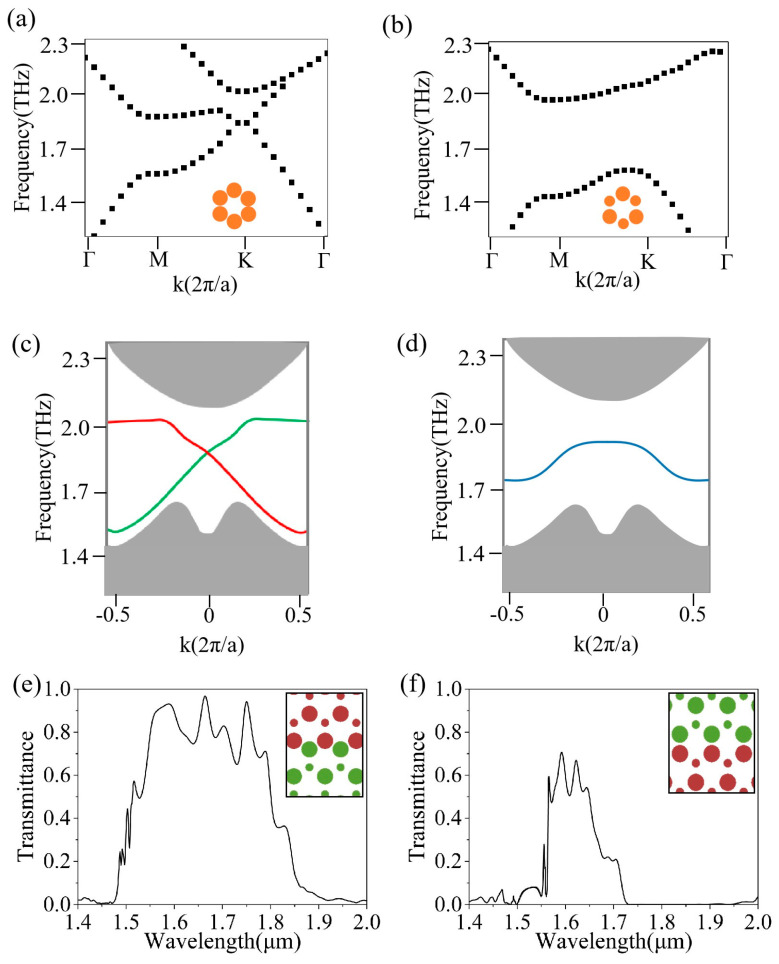
(**a**) The band diagram of germanium PCs with a honeycomb lattice structure. The radius of the orange circle is 75 nm; (**b**) Band diagram of germanium VPC1. The radius of the larger circle is 108 nm, while the radius of the smaller circle is 30 nm. (**c**,**e**) are band structures and transmission spectra of zigzag edge states. The edge state plots in (**c**) in red and green correspond to the LCP and RCP light; (**d**,**f**) are band structures and transmission spectra of the beard edge states. The edge state plot in (**d**) in blue corresponds to circularly polarized light. The red and green circles in (**e**,**f**) correspond to VPC1 and VPC2.

Numerical simulations were performed using the Finite-Difference Time-Domain method (commercial Lumerical FDTD 2020 software). The band structures of the honeycomb lattice photonic crystal and valley photonic crystal 1 (VPC1) were calculated under transverse electric (TE) polarization, as shown in Figure 2a,b, respectively. When the cylindrical radius in the honeycomb lattice is 81 nm, the band diagram reveals a Dirac point within the optical communication range located at a wavelength of 0.2536 *a*/*λ* (1617 nm), as shown in Figure 2a. VPC1 consists of air cylinders arranged in a honeycomb lattice within a germanium substrate, with a lattice constant *a* of 0.41 µm. The unit cell of VPC1 contains three large air holes and three small air holes, which break the spatial inversion symmetry of the honeycomb structure, as shown in Figure 2b. When the radii of the air cylinders in three directions are set to a larger radius *r*_1_ of 108 nm and the radii in the other three directions are set to a smaller radius *r*_2_ of 30 nm, the structural rotational symmetry is reduced from C_6v_ to C_3v_. This opens a band gap at the Dirac point (*K* point), resulting in a photonic band gap ranging from 0.2270 *a*/*λ* to 0.2766 *a*/*λ* (1482 nm to 1806 nm). The width of this photonic bandgap determines the bandwidth of the topological edge states, providing the theoretical foundation for the wide-bandwidth unidirectional optical transmission.

Valley photonic crystal 2 (VPC2) is obtained by mirroring VPC1. By interfacing these two mirror-symmetric VPCs, different types of edge states are formed, namely the zigzag edge and the bearded edge, as shown in Figure 2c–f. VPC1 and VPC2 can be combined to form a topological waveguide, so that the edge states (shown in the band diagram in Figure 2c by the red and green lines, corresponding to the LCP and RCP light) can propagate along the direction to the “locking” valley. The figures indicate that passbands appear in both edge-state structures, demonstrating that both can support valley-momentum-locked optical transport and that their working bandwidths are within the optical communication band (details are shown in Supplementary Materials, Section S2). Comparing the transmission efficiency and bandwidth of the two edge states reveals that the zigzag edge exhibits higher transmission (greater than 0.7) and a broader bandwidth of 1545 nm to 1792 nm (247 nm), with a peak transmission of up to 0.97, as shown in Figure 2e,f. Therefore, the zigzag edge state was selected for the design of the topological ring resonator pressure sensor. The details of the comparison of zigzag edge states and beard edge states are shown in Supplementary Materials, Section S3.

**Figure 3:**
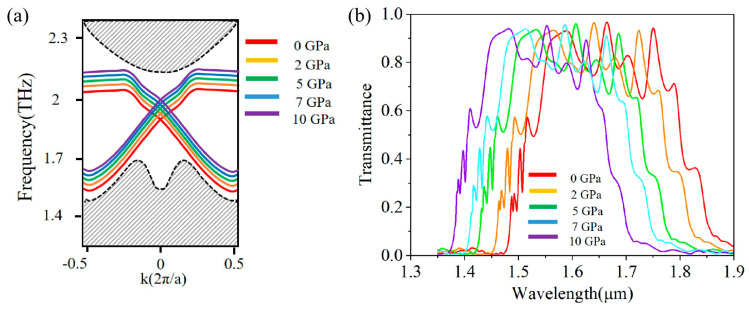
(**a**) The band diagram of the edge state structure under different pressures, the shaded area represents the bulk states; (**b**) The transmittance of the zigzag waveguide under different pressures.

By varying the refractive index of the germanium-based valley photonic crystal to simulate different applied pressures, the bands of the zigzag edge state were obtained. As external pressure increases, the central frequency of the photonic band for the zigzag topological edge state shifts from 1563.5 nm to 1523 nm, as shown in Figure 3a. The working bandwidth of the edge-state structure shifts from 1422 nm to 1705 nm at 0 GPa to 1399 nm to 1647 nm at 10 GPa, indicating a shift toward higher frequency in the bandwidth. According to the transmission spectrum in Figure 3b, the working bandwidth of the structure gradually narrows with increasing pressure, decreasing from 211 nm to 192 nm. Although the working bandwidth has narrowed, the overall transmittance remains above 0.8, indicating that this topological edge state meets the requirements for designing pressure sensors. Regarding the oscillatory phenomenon observed at the top of the transmission spectrum bandwidth in Figure 3b, this phenomenon primarily stems not from a strict Fabry-Perot (F-P) cavity, but rather from a weak F-P effect induced by minor mismatches in impedance between the coupling region of the topological waveguide and the ring resonator, as well as at the input/output ports. This induces a small amount of back reflection that interferes with the forward-propagating waves, resulting in the observed low-frequency oscillations (ripples) in the transmission spectrum, which could be further optimized in future designs by improving mode matching. Nevertheless, this does not affect the validation of the device principle proposed herein or the demonstration of its core performance.

## 3. Analysis and Discussion

**Figure 4:**
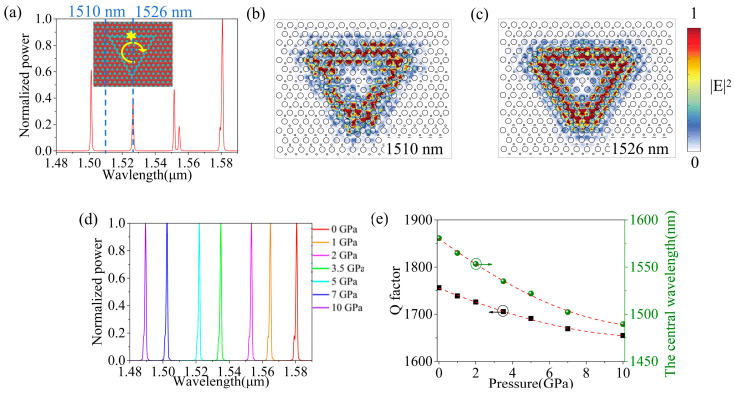
(**a**) Normalized power and structural schematic diagram of the germanium topological ring resonator without pressure, where the stars represent the RCP light, and the direction of the arrows indicates the transmission direction of the light along the resonator; (**b**) The electric field intensity distribution of the topological ring resonator at the wavelength of 1510 nm; (**c**) The electric field intensity distribution of the topological ring resonator at the wavelength of 1526 nm; (**d**) Normalized power of resonant peaks at different pressures; (**e**) The variation of the resonant peak quality factor and the central wavelength of the resonant peak with the change of pressure, where the green balls and the black squares represent the central wavelength and the Q factor, respectively. The red dashed lines are the fitted curves.

Based on the robustness of the topological edge state structure in 60° and 120° bent waveguides, we designed a triangular ring resonator composed of germanium-based VPCs. The total length of the ring is 27*a*. The interior and exterior of the ring resonator correspond to two regions with different topological properties (VPC1 and VPC2), as shown in Figure 4a. The basic unit of the triangular topological ring resonator is a structure comprising VPC1 and VPC2, which consist of circular air holes arranged in a germanium slab. In the figure, the asterisk indicates the location of the light source, and the arrows indicate the direction of light propagation within the ring resonator.

The normalized power spectrum distribution of an RCP light source within the topological ring resonator was simulated. As can be seen from Figure 4a, multiple sharp peaks appear in the power spectrum of the triangular ring resonator, and these peaks are uniformly distributed within the operational bandwidth of the ring. This indicates that the resonator couples light at specific wavelengths into its modes. Four resonance peaks are uniformly distributed within the range of 1480 nm to 1590 nm. Among them, the resonance peak at 1580 nm exhibits high transmittance. Its Q-factor is calculated using the formula:

*Q* = *λ_center_*/*FWHM*, (2)

where *FWHM* is the full width at half maximum and *λ_center_* is the central wavelength of the resonance peak. The calculated Q-factor at the 1580 nm wavelength is 1755.6 [17]; the details are shown in Supplementary Materials, Section S4. From Figure 4b,c, it can be observed that light at 1526 nm is confined and propagates along the topological ring resonator, indicating the presence of a resonant mode at this wavelength. In contrast, light at 1510 nm is not effectively bound to the edge state for propagation, indicating the absence of a resonant mode.

To explore the coupling of light waves in germanium-based topological ring resonators under different pressures, we simulated the resonant peak with the highest power at 1580 nm as the target wavelength, as shown in Figure 4d. The resonant peak shifts to higher frequencies with increasing applied pressure. As shown in Figure 4d, the resonance peak shifts to higher energy with increasing external pressure. Figure 4e shows that the central wavelength of the resonant peak (initially at 1580 nm) moves toward shorter wavelengths as the external pressure increases. Within this pressure range, the *Q* factor remains between 1650 and 1750, gradually decreasing with increasing pressure. The *Q* factors shown in Figure 4e all refer to the resonator’s intrinsic *Q* Factor.

**Figure 5:**
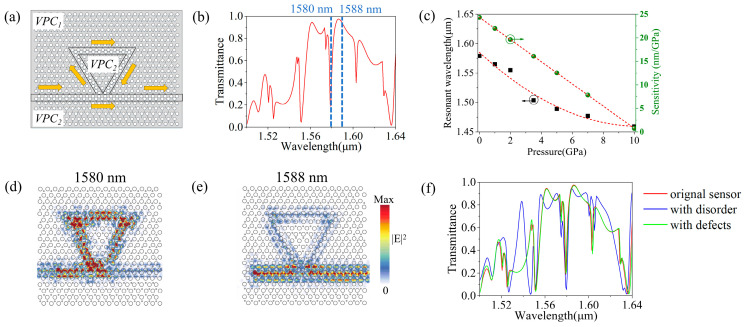
(**a**) Schematic diagram of the structure of the topological ring resonator pressure sensor, where the yellow arrow indicates the transmission direction of RCP light in the sensor; (**b**) Transmittance spectrum of the topological pressure sensor. (**c**) The variation of the resonant wavelength and sensitivity of the sensor under different pressures, where the red line, the green ball, and the black square represent the fitting curve, the sensitivity, and the resonant wavelength, respectively. The electric field intensity distributions at the notch peak wavelength of 1580 nm and the transmittance peak of 1588 nm in (**d**–**f**) Transmittance spectrum contrast of the proposed topological sensor in the presence of disorder or defects.

A pressure sensor composed of the topological ring resonator and a topological straight waveguide was designed. The transmission spectrum under zero applied pressure is shown in Figure 5a,b. In addition to the significant decrease in the resonant peak, the transmission remains above 0.6 across other wavelengths, reaching a maximum of 0.97. The shift of the resonance dip (initially at 1580 nm) under different pressures was simulated for the topological pressure sensor, as shown in Figure 5c. Under applied pressures of 0 GPa, 2 GPa, 5 GPa, 7 GPa, and 10 GPa, the notch wavelengths are 1580 nm, 1555 nm, 1489 nm, 1477 nm and 1459 nm, respectively. Comparing the unidirectional transmission curve of the germanium-based topological ring resonator pressure sensor (Figure 5a) with that of the zigzag straight waveguide (Figure 2e), the forward transmission of the sensor remains largely above 0.8, with a peak exceeding 0.95. This indicates that the sensor structure achieves high-transmission sensing via resonance wavelength shifts without significantly compromising the overall forward transmission. When the pressure is less than 3 GPa, the position of the resonance peak in the entire sensor output spectrum remains essentially unchanged, as shown in Figure 5c in the manuscript. At a pressure of 3 GPa, the deformation of the entire germanium plate increases to 6.6 nm. Consequently, the structure’s thickness affects the operating bandwidth, resulting in a shift of approximately 10 nm in the resonance peak position compared to the same pressure reported in the original paper. Furthermore, within the pressure-sensing range of 0 to 10 GPa, the resonant wavelength shifts from 1580 nm to 1459 nm, with a detection sensitivity of 24.34 nm/GPa to 0.74 nm/GPa for the topological ring resonator sensor, which is higher than the other designs [10,12,17]. Throughout the process, the germanium material’s refractive index changes with applied pressure, thereby tuning the triangular resonator’s optical properties and shifting the resonance dip. The shift of the dip position with increasing pressure is consistent with the direction of the resonance peak shift. These results demonstrate that the proposed pressure sensor effectively performs pressure sensing within the optical communication band. By monitoring the shift in the resonance dip wavelength, the applied pressure in the range of 0–10 GPa can be determined, thereby fulfilling the purpose of pressure sensing.

To examine the coupling between the triangular topological ring resonator and the straight waveguide, the electric field distributions at both the resonance dip wavelength and a high-transmission wavelength were simulated, as shown in Figure 5d,e. The results demonstrate the optical transmission along the topological edge states. In Figure 5d, at the resonance dip wavelength of 1580 nm (where a resonant mode exists), most of the power is coupled into and confined within the ring resonator, minimizing propagation along the straight waveguide. This is because when the resonant wavelength matches the triangular ring resonator’s mode, a high-*Q* resonant mode forms within the resonator, creating local high-energy regions, as shown in the field diagram. Due to the valley-momentum-locking effect, the electric field remains well confined within the ring, even at sharp bends, with minimal scattering loss, indicating effective photon confinement. In contrast, the light passes the ring almost entirely and is directly output to the right port in the straight waveguide at 1588 nm (Figure 5e). Backscattering from the source is effectively suppressed owing to the valley-momentum locked properties of the topological edge states. Furthermore, the topological ring resonator based on valley photonic crystals inherently benefits from its “defect-immune” and unidirectional transmission characteristics, which theoretically protect the edge states and resonant modes from certain types of perturbations (details are shown in Supplementary Materials, Section S5).

At the same time, the performance of other photonic crystal pressure sensors in the optical communication range was compared, as shown in Table 1. As shown in Table 1, the structure proposed in this paper features a simpler design, smaller device dimensions, a wider operating bandwidth, and higher sensing sensitivity. Furthermore, the topological photonic crystal edge states exhibit unidirectional transmission. Its performance is significantly superior to that of other PC pressure sensors in these areas, making it suitable for pressure-sensor applications in photonic integration.

## 4. Conclusions

In summary, we theoretically designed a pressure sensor structure based on a germanium-based valley photonic crystal ring resonator and achieved optical sensing in the optical communication band. Due to the change in the material properties of germanium under the application of pressure, the resonance mode of the germanium-based topological resonant ring will also shift. By monitoring the shift of a specific resonance peak in the triangular ring resonator, external pressure is detected. This structure operates over a pressure range of 0–10 GPa, with resonance wavelengths spanning 1459-1580 nm. Different resonance wavelengths correspond to different pressures, yielding a maximum detection sensitivity of 24.34 nm/GPa. The topological ring resonator pressure sensor has a compact footprint of 7.5 μm × 6.5 μm, and its output transmission spectrum exhibits a maximum unidirectional transmittance of 0.97. Theoretically, this enables high-transmittance pressure sensing within the optical communication range. Combined with current CMOS processing technology, the germanium-based topological ring resonator pressure sensor structure can further miniaturize integrated pressure sensors and enhance measurement sensitivity, offering a novel approach for optical sensing. It enables real-time, in situ pressure monitoring in some key areas: (1) monitoring internal pressure in optical communication components for fault warning [39] and (2) ensuring the safety of optical modules in extremely high-pressure environments like deep-sea or downhole applications [40].

## Figures and Tables

**Table 1:** Comparison of structural parameters and performance of different PC pressure sensors.

Type	Footprint	Material	Sensitivity	Peak	Bandwidth	Unidirectional Transmission
Polymer-Based PC Pressure Sensor (2024) [17]	11 × 8.6 μm^2^	Polymer	21 nm GPa^−1^	1.744 μm	Single wavelength	No
One-Dimensional Symmetric Defect PC Pressure Sensor (2023) [10]	None	Superconductor and GaAs	2.6 nm GPa^−1^	1.0508 μm	Single wavelength	No
PC Sensor with Dual Microcavity Coupled Waveguides (2023) [12]	15 × 7 μm^2^	InAs	26.1 nm GPa^−1^	1.8395 μm	180 nm	No
This work	7.5 × 6.5 μm^2^	Ge	24.34 nm GPa^−1^	1.650 μm	247 nm	Yes

## Data Availability

The data that support the findings of this study are available from the corresponding author upon reasonable request.

## Supplementary Materials

The following supporting information can be downloaded at: https://www.mdpi.com/article/10.3390/s26020659/s1, S1. Potential effects of pressure on germanium-based topological ring resonators. S2. Unidirectional transmission performance of zigzag structures. S3. Comparison of zigzag edge states and beard edge states. S4. Discussion on the Q-factor in Ring Resonator Sensors. S5. Potential manufacturing tolerance of germanium-based topological ring resonator pressure sensors. References [41,42] are cited in Supplementary Materials file.

## References

[1] Venkateswara R.K., Indira B., Basavaprasad, Dudla P., Srinivas T. (2021). A high Q-factor photonic crystal microring-resonator based pressure-sensor. Photonics Nanostructures Fundam. Appl..

[2] Kolli V.R., Basavaprasad, Bahaddur I., Talabattula S. (2021). A high sensitive photonic crystal Mach-Zehnder-Interferometer based pressure-sensor. Results Opt..

[3] Liang H., Wang J., Zhang L., Liu J., Wang S. (2022). Review of optical fiber sensors for temperature, salinity, and pressure sensing and measurement in seawater. Sensors.

[4] Xu S., Li X., Wang T., Wang X., Liu H. (2023). Fiber Bragg grating pressure sensors: A review. Opt. Eng..

[5] Li C., Zhang C., Yang L., Guo F. (2022). Silicon-on-Insulator optical waveguide pressure sensor based on Mach-Zehnder Interferometer. Micromachines.

[6] Yang M., Zhu Y., Ren J. (2024). Hourglass-shaped fiber-optic Mach-Zehnder interferometer for pressure sensing. Opt. Fiber Technol..

[7] Bahadoran M., Aghili A., Noorden A.F.A. (2022). Micro-opto-mechanical pressure sensor via ring resonator-based Mach-Zehnder Interferometer. Eur. Phys. J. Plus.

[8] Lai W., Li B., Fu S., Lin Y. (2023). Tunable MEMS-based terahertz metamaterial for pressure sensing application. Micromachines.

[9] Dharchana T., Sivanantharaja A., Selvendran S. (2017). Design of pressure sensor using 2D photonic crystal. Adv. Nat. Appl. Sci..

[10] Hu H., Chen X., Zhao M., Wang L., Fang M., Zhao D. (2023). Pressure sensing of symmetric defect photonic crystals composed of superconductor and semiconductor in low-temperature environment. Crystals.

[11] Vahid F., Zoheir K., Mehdi H. (2024). Sensitivity and quality factor improvement of photonic crystal sensors by geometrical optimization of waveguides and micro-ring resonators combination. Sci. Rep..

[12] Zouache T., Hocini A. (2023). A 2D photonic crystal indium arsenide based with dual micro-cavities coupled to a waveguide as a platform for a high sensitivity pressure sensor. Opt. Quantum Electron..

[13] Zaky Z.A., Al-Dossari M., Sharma A., Aly A.H. (2023). Effective pressure sensor using the parity-time symmetric photonic crystal. Phys. Scr..

[14] Norouzi S., Fasihi K. (2022). Realization of pressure sensor based on a GaAs-based two dimensional photonic crystal slab on SiO_2_ substrate. J. Comput. Electron..

[15] Zouache T., Hocini A., Harhouz A., Mokhtari R. (2017). Design of pressure sensor based on two-dimensional photonic crystal. Acta Phys. Pol. A.

[16] Gowda R.B., Sharan P., Saara K. (2023). 1-Dimensional silicon photonic crystal pressure sensor for the measurement of low pressure. Results Opt..

[17] Lotfi Hayaei A. (2024). Design, simulation, and optimization of a polymer-based photonic crystal pressure sensor. Opt. Quantum Electron..

[18] Cheng Q., Wang S., Lv J., Liu N. (2022). Topological photonic crystal biosensor with valley edge modes based on a silicon-on-insulator slab. Opt. Express.

[19] Arora S., Bauer T., Barczyk R., Verhagen E., Kuipers L. (2021). Direct quantification of topological protection in symmetry-protected photonic edge states at telecom wavelengths. Light: Sci. Appl..

[20] Chen Y., He X.T., Cheng Y.J., Qiu H.Y., Feng L.T., Zhang M., Dai D.X., Guo G.C., Dong J.W., Ren X.F. (2021). Topologically protected valley-dependent quantum photonic circuits. Phys. Rev. Lett..

[21] Chen X., Zhao F., Chen M., Dong J. (2017). Valley-contrasting physics in all-dielectric photonic crystals: Orbital angular momentum and topological propagation. Phys. Rev. B.

[22] Kumar A., Gupta M., Pitchappa P., Tan Y.J., Wang N., Singh R. (2022). Topological sensor on a silicon chip. Appl. Phys. Lett..

[23] Gu L., Yuan Q., Zhao Q., Ji Y., Liu Z., Fang L., Gan X., Zhao J. (2021). A topological photonic ring-resonator for on-chip channel filters. J. Light. Technol..

[24] Lai K., Ma T., Bo X., Analage S., Shvets G. (2016). Experimental realization of a reflections-free compact delay line based on a photonic topological insulator. Sci. Rep..

[25] Mehrabad M.J., Foster A.P., Dost R., Clarke E., Patil P., Farrer I., Heffernan J., Skolnick M., Wilson L. (2020). A semiconductor topological photonic ring resonator. Appl. Phys. Lett..

[26] Barik S., Karasahin A., Mittal S., Waks E., Hafezi M. (2020). Chiral quantum optics using a topological resonator. Phys. Rev. B.

[27] Lu L., Joannopoulos J.D., Soljačić M. (2014). Topological photonics. Nat. Photonics.

[28] Ezawa M. (2013). Topological Kirchhoff law and bulk-edge correspondence for valley Chern and spin-valley Chern numbers. Phys. Rev. B.

[29] Wu M., Yang Y., Fei H., Lin H., Han Y., Zhao X., Chen Z. (2022). Unidirectional transmission of visible region topological edge states in hexagonal boron nitride valley photonic crystals. Opt. Express.

[30] Wu M., Yang Y., Fei H., Lin H., Zhao X., Kang L., Xiao L. (2022). On-chip ultra-compact hexagonal boron nitride topological ring-resonator in visible region. J. Light. Technol..

[31] Yang Z., Fei H., Wu M., Lin H. (2025). Topological ring resonator for refractive index sensing at telecommunication wavelength. Micro Nanostructures.

[32] Fei H., Wu M., Lin H., Yang Y., Xiao L. (2024). A tunable hexagonal boron nitride topological optical delay line in the visible region. Chin. Opt. Lett..

[33] Zhang Q., Liu Z., Wu J., Sun P., Zhang H. (2026). Design and performance analysis of a hybrid flexible pressure sensor with wide linearity and high sensitivity. Sensors.

[34] Ke A., Li C., Mai Z. (2025). Simulation for regulatable assembly of large-scale photonic crystal: Application in flexible pressure sensor with visual sensing. Chem. Eng. Sci..

[35] Wu M., Bai J., Zhao X. (2025). Design of pressure sensors based on the Mach-Zender interferometer of the valley photonic crystal. Opt. Commun..

[36] Mishra C.S. (2025). Design and Analysis of 2-D Photonic honeycomb structure for application of pressure sensor. IEEE Trans. Instrum. Meas..

[37] Mohammed Y., Zhang K., Heissler S., Baumgart H., Elmustafa A. Phase transformation in germanium-on-insulator (GEOI) films using raman spectroscopy and nanoindentation. Proceedings of the 237th ECS Meeting.

[38] Oliver D.J., Bradby J.E., Williams J.S., Swain M.V., Munroe P. (2008). Thickness-dependent phase transformation in nanoindented germanium thin films. Nanotechnology.

[39] Dinodiya S., Bhargava A. (2022). A comparative analysis of pressure sensing parameters for two dimensional photonic crystal sensors based on Si and GaAs. Silicon.

[40] Sherawat V., Bokolia R., Sinha R.K. (2024). Pressure-dependent bandgap characteristics in photonic crystals with sensing applications. J. Opt..

[41] Gill G.S., Jones C., Tripathi D., Keating A., Putrino G., Silva K., Faraone L., Martyuniuk M. (2022). Correction to: Mechanical properties of thermally evaporated germanium (Ge) and barium fluoride (BaF_2_) thin-films. MRS Commun..

[42] Yuan Q., Li S., Zhou L., He D. (2022). Phase-pure ST12 Ge bulks through secondary pressure induced phase transition. Solid State Commun..

