# Supplementary material for: Topological Photonic Crystal Ring Resonator Pressure Sensor in the Optical Communication Range
Source: Sensors (Basel). 2026 Jan 19;26(2):659. doi: 10.3390/s26020659 (PMC12845562; doi:10.3390/s26020659)
Supplement: Supplementary file 1 [file sensors-26-00659-s001.zip › sensors-4077429-supplementary.pdf]

sensors

MDPI

Article

Topological Photonic Crystal Ring Resonator Pressure Sensor in the Optical Communication Range

Min Wu [1,2,†], Zhuoxin Yang [1,†], Hongming Fei [1,*] and Han Lin [3,*]

1 College of Physics and Optoelectronics, Taiyuan University of Technology, Taiyuan 030024, China; wumin2@sxgkd.edu.cn (M.W.); yzx990921@163.com (Z.Y.)
2 College of Information Engineering, Shanxi Vocational University of Engineering Science and Technology, Jinzhong 030619, China
3 Centre for Atomaterials and Nanomanufacturing, School of Science, RMIT University, Melbourne, Victoria 3000, Australia
* Correspondence: feihongming@tyut.edu.cn (H.F.); han.lin2@rmit.edu.au (H.L.)
† These authors contributed equally to this work.

S1. Potential effects of pressure on germanium-based topological ring resonators

We confirm that the 220 nm thick single-crystalline Ge film [ref. 15 in the manuscript] in our design is mechanically adequate for the target operating pressure range of 0–10 GPa. The key rationale lies in the fundamental difference in mechanical properties between nanoscale films and their bulk counterparts. Experimental studies have shown that when Ge is scaled down to nanoscale thicknesses (e.g., 220 nm), it exhibits a significant " softening " effect, characterized by reduced hardness and elastic modulus. Specifically, a single-crystalline Ge film approximately 220 nm thick has a hardness of about 8–10 GPa. This hardness value is higher than the maximum operating pressure of 10 GPa for our sensor, providing a fundamental safety margin [1]. More importantly, the fracture mechanism of ultra-thin Ge films changes. For films in the hundred-nanometer thickness range, the dominant deformation mechanism under pressure becomes pressure-induced phase transformations and other controllable microstructural rearrangements, rather than the direct propagation of macroscopic brittle cracks typical in bulk materials. This allows the film to accommodate high pressure up to 12 GPa [2] in a more ductile manner, avoiding catastrophic failure. Consequently, setting the sensor's upper limit at 10 GPa is reasonable, as this value falls within the allowable range defined by the film's hardness, and its nanostructured nature grants it superior pressure adaptability.

S2. Unidirectional transmission performance of zigzag structures

Regarding whether the zigzag structure in the article enables unidirectional transmission, we first assess this using the band structure. This structure exhibits valley-momentum-locked transmission and unidirectional conduction. We calculated the transmittance spectra of RCP light propagating in the straight waveguide (Figure 2e in the manuscript) along the forward (right) and backward (left) directions. The transmission contrast is defined as $C=|(T_F-T_B)/(T_F+T_B)|$, the forward transmittance T_F, the backward transmittance T_B and the transmittance contrast C, as shown in Figure S1. The electric field distribution reveals that left-circularly/right-circularly polarized light exhibits a spin-locked valley coupling effect with the edge states. Under this combination of edge states, the transmission spectrum shows a forward transmittance exceeding 0.8 and a reverse transmittance below 0.05; the transmittance contrast C is higher than 0.9, achieving

 https://doi.org/10.3390/s26020659

unidirectional transmission of right-handed polarized light. The forward (right) and backward (left) transmittances are T_F and T_B, respectively.

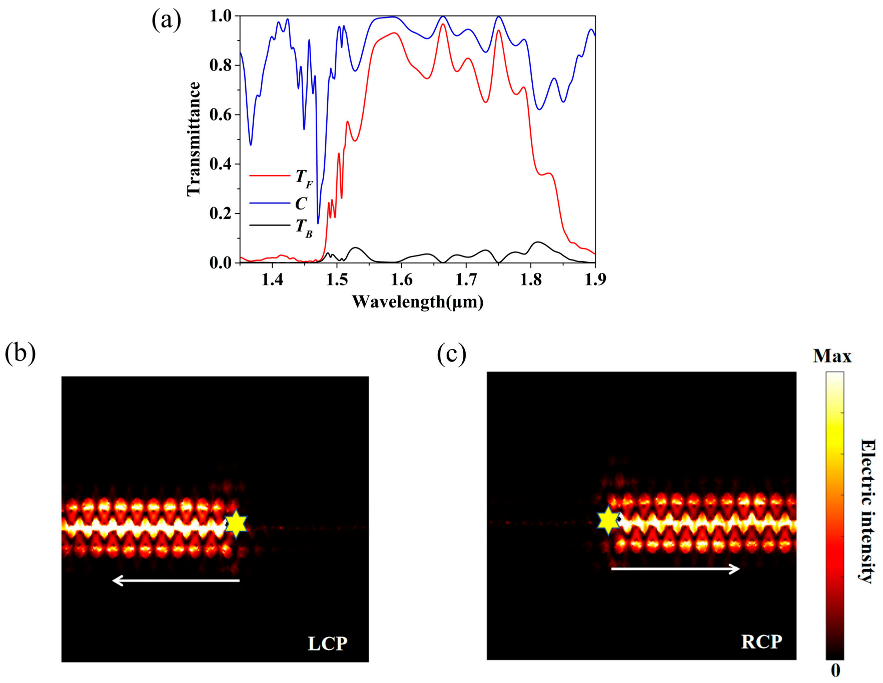

Figure S1. (a) The forward transmittance T_F, the backward transmittance T_B, and the transmittance contrast C of RCP light. The electric field intensity distributions of RCP (b) and LCP (c) light, where the stars represent the LCP/RCP light, and the direction of the white arrows indicates the transmission direction of the light along the waveguides.

S3. Comparison of zigzag edge states and beard edge states

In valley photonic crystals (VPCs), the direction of energy flow and the mode profile (i.e., mode localization) of the edge states are determined jointly by their topological Chern number and the boundary geometry. For the VPCs we employ, although both the zigzag and bearded edges support unidirectional topological boundary states, their intrinsic electromagnetic modes exhibit significant differences in localization properties. Theoretical calculations and simulations indicate that, under identical structural parameters, the optical field energy of the zigzag edge state is more tightly confined near the interface. This signifies that the zigzag edge state possesses a stronger field confinement capability. The optical field is more strictly confined within the ring resonator, resulting in less energy loss via radiation from the waveguide bends.

Therefore, our choice of the zigzag edge state is based not only on its wider unidirectional transmission bandwidth and higher transmittance in the communication band as mentioned in the Abstract and shown in Figure 2 but also, on a deeper physical level, on its superior mode localization property. This property provides a more solid foundation for constructing a low-loss, high-Q topological ring resonator, which is a critical design consideration for achieving high-sensitivity pressure sensing (such as the high sensitivity of 24.34 nm/GPa reported in the Abstract).

S4. Discussion on the Q-factor in Ring Resonator Sensors

The critical distinction between the simulation setups in the inset of Figure 4a (internal point source excitation) and Figure 5 of the manuscript (waveguide-coupled excitation), as well as the conceptual difference between the Intrinsic Q Factor and the

Loaded Q Factor, is explained as follows. This is essential for accurately interpreting the pressure-dependent Q-factor data presented in Figure 4e of the manuscript.

The Q factors shown in Figure 4e of the manuscript all refer to the resonator's intrinsic Q Factor (Q_i), not the loaded Q factor. In our pressure-sensing study, we focus on how pressure alters the resonator's own physical properties (e.g., changing the refractive index via the photoelastic effect, thereby affecting the mode distribution and losses). Analyzing the intrinsic Q factor allows us to exclude variables and complexities potentially introduced by external coupling conditions (e.g., waveguide-resonator gap distance), thus revealing the intrinsic impact of pressure on the resonator's performance more directly and purely. This provides the most fundamental parameter for assessing the sensor's stability and signal detectability under high pressure. The Q factor was calculated using the formula $Q=f_0/\Delta f$, where f_0 is the resonant frequency and Δf is the full width at half maximum of the resonance peak. The Q value obtained usings this method corresponds directly to Q_i, as it simulates the free decay or intrinsic linewidth of the resonant mode in the absence of external waveguide coupling.

Therefore, the vertical axis label of Figure 4e should be more precisely labeled as "Intrinsic Q Factor". It systematically demonstrates the combined variations in the resonator's radiation and material losses across the 0–10 GPa pressure range. In comparison, the structure shown in Figure 5 is primarily used to demonstrate the device's complete transmission spectrum in its practical operating state (including coupling effects).

S5. Potential manufacturing tolerance of germanium-based topological ring resonator pressure sensors

We also conducted numerical simulations to quantitatively demonstrate the minor influence of structural disorder on the displacement of the resonant peak, as shown in Figure S2. When the resonator structure shows disorder or defects, the shift in the resonant peak is relatively small, and the overall maximum transmittance remains above 0.9. In our design, the topological ring resonator based on valley photonic crystals inherently benefits from its "defect-immune" and unidirectional transmission characteristics, which theoretically protect the edge states and resonant modes from certain types of perturbations.

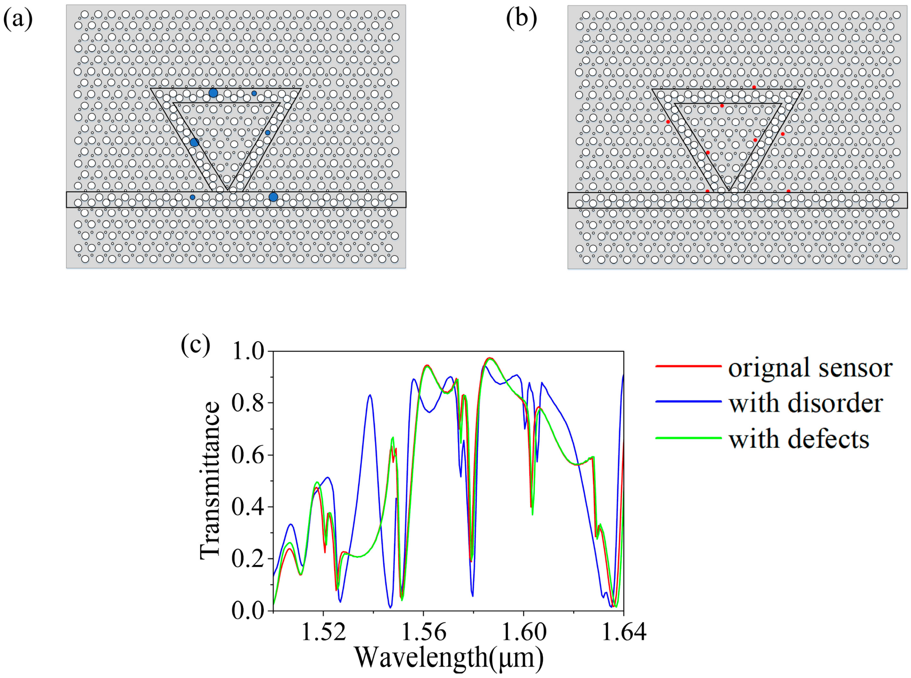

https://doi.org/10.3390/s26020659

Figure S2. (a) and (b) are the schematic diagram of the structure of the topological ring resonator pressure sensor in the presence of disorder or defects, where the blue circles represent disorder (the radius of the hole becomes larger or smaller) and the red circles represent defects (the hole disappeared); (c) transmittance spectrum contrast of the proposed topological sensor in the presence of disorder or defects.

References

1. Gill, G.S.; Jones, C.; Tripathi, D.; Keating, A.; Putrino, G.; Silva, K.;Faraone, L; Martyuniuk, M. Correction to: Mechanical properties of thermally evaporated germanium (Ge) and barium fluoride (BaF_2) thin-films. *MRS Commun.* **2022**, *12*, 284.
2. Yuan, Q.; Li, S.; Zhou, L.; He, D. Phase-pure ST12 Ge bulks through secondary pressure induced phase transition. *Solid State Commun.* , **2022**, 348-349, 114742

Disclaimer/Publisher's Note: The statements, opinions and data contained in all publications are solely those of the individual author(s) and contributor(s) and not of MDPI and/or the editor(s). MDPI and/or the editor(s) disclaim responsibility for any injury to people or property resulting from any ideas, methods, instructions or products referred to in the content.

https://doi.org/10.3390/s26020659
